# Impact of gonadotropin genetic profile and ovarian reserve on controlled ovarian stimulation: data from prospective cohort of the GENACOS trial

**DOI:** 10.3389/fendo.2025.1601803

**Published:** 2025-08-22

**Authors:** Alessandro Conforti, Daniele Santi, Adolfo Allegra, Mario Mignini Renzini, Angelo Marino, Claudio Brigante, Roberta Iemmello, Valeria Stella Vanni, Agnese Rebecchi, Laura Privitera, Samantha Sperduti, Livio Casarini, Ilma Floriana Carbone, Manuela Simoni, Carlo Alviggi, Enrico Papaleo

**Affiliations:** 1 Department of Neuroscience, Reproductive Science and Odontostomatology, University of Naples Federico II, Naples, Italy; 2 Unit of Endocrinology, Department of Biomedical, Metabolic and Neural Sciences, University of Modena and Reggio Emilia, Modena, Italy; 3 Reproductive Medicine Unit, ANDROS Day Surgery Clinic, Palermo, Italy; 4 Biogenesi Reproductive Medicine Centre, Istituti Clinici Zucchi, Monza, Italy; 5 Gynecology/Obstetrics Unit, IRCCS San Raffaele Scientific Institute, Milan, Italy; 6 Istituto Eugyn, Modena, Italy; 7 Center for Genomic Research, University of Modena and Reggio Emilia, Modena, Italy; 8 Unit of Obstetrics, Department of Woman, Child and Neonate, Mangiagalli Center, Fondazione IRCCS Ca’ Granda Ospedale Maggiore Policlinico, Milan, Italy; 9 Department of Public Health, University of Naples Federico II, Naples, Italy

**Keywords:** polymorphisms, pharmacogenomic, ovarian stimulation, IVF/ICSI, ART, genetic variants

## Abstract

**Introduction:**

Several studies indicate that a specific genotype profile could influence ovarian sensitivity to exogenous gonadotropin. However, most of the previous studies were observational and retrospective and thereby more prone to bias. The aim of this study was to evaluate the impact of gonadotropin single nucleotide polymorphisms (SNPs) on the outcomes of *in-vitro* fertilization (IVF) in infertile patients undergoing their first ovarian stimulation (OS) cycle.

**Method:**

A multicenter, longitudinal, prospective, interventional cohort study was carried out in four clinical centers of medically assisted reproduction from August 2016 to November 2018. Only expected normo-responder women, estimated through standardized-computerized antral follicle count (AFC), stimulated with a fixed 150 IU daily dose of recombinant follicle-stimulating hormone (FSH), were included. The study population consisted of infertile normo-gonadotropic patients, aged between 34 and 39, at their first OS, with normal ovarian reserve (AFC between 8 and 16) measured with 3D automated ultrasonography and undergoing standardized OS protocol.

**Results:**

One hundred nineteen patients were enrolled, and the following five SNPs were studied (*FSHR* c.-29G>A, FSHR p.N680S, *FSHB* c.-211G>T, LHCGR p.S312N, and LHβ “V-LH” p.W8R). Separate and multivariate analysis of investigated polymorphisms did not show any statistical impact on the number of oocytes retrieved. However, adopting an overdominant model, heterozygosis of FSHR p.N680S SNP was associated with significantly lower duration of OS compared with homozygotic women. Considering LHCGR p.S312N polymorphism, N allele carriers required a longer duration of OS in the codominant, dominant, and log-additive models. Multivariate analysis revealed that specific genotype combinations could affect the ovarian sensitivity. A significantly higher follicle-to-oocyte index (FOI) was observed when the S or N allele of both FSHR p.N680S and LHCGR p.S312N were combined (S allele combination: difference 0.18, CI 95% 0.04–0.33, *p* = 0.011; N allele combination: difference 0.18, CI 95% 0.01–0.34, *p* = 0.037; N allele combination).

**Discussion:**

Based on our results, the combination of specific genetic variants could impact ovarian sensitivity to gonadotropin. This research adds to the controversy in the literature regarding the effect of genetic variants in IVF and ovarian response.

## Introduction

The crucial stage in assisted reproductive technologies (ART) is ovarian stimulation (OS), wherein the ovaries are stimulated primarily to acquire the highest number of oocytes (1). Research indicates that the live birth rate consistently rises when retrieving between 8 and 15 oocytes in fresh ART cycles (2). Moreover, in combined fresh and frozen cycles, an amount of more than 15 oocytes notably enhances the likelihood of achieving at least one successful delivery (3). Instead, low or poor responders are usually associated with a low number of oocytes retrieved at the end of stimulation. According to European Society of Human Reproduction and Embryology (ESHRE) guidelines, poor responders are those with less than four oocytes retrieved at the end of the OS phase (4). This concept was stressed even in the last Patient-Oriented Strategies Encompassing IndividualizeD Oocyte Number (POSEIDON) criteria, in which ovarian sensitivity and age-related embryo/blastocyst aneuploidy rate were considered to be crucial for poor responsiveness (5, 6). If no previous ART cycles have been performed, the prediction of OS outcomes is based on both women’s age and ovarian reserve markers, such as antral follicle count (AFC) and anti-Mullerian hormone (7). However, AFC exhibits several limitations, mainly due to inter-operator interpretation issues (8–10). To address this drawback, the use of ultrasonographic automated three-dimensional (3D) follicular count (11, 12) in conjunction with automatic volume calculation (AVC) has been proposed as a reliable alternative to standard 2D evaluation. Many studies demonstrated that AVC significantly enhances inter-observer reliability of AFC, offering highly accurate assessments of both number and size of antral follicles when compared to traditional 2D evaluation (13, 14). Indeed, if appropriately standardized across centers, AVC may be used as a centralized test to assess ovarian reserve.

Insights from the past decade suggest that women sharing similar demographic, anthropometric, and gonadotropin level profiles may exhibit markedly different ovarian responses (15, 16), even when possessing similar ovarian reserve levels. In other words, the “sensitivity” of follicles to exogenous gonadotropins may vary among infertile women (17, 18). For example, two women with the same AFC may have different follicular output rates (FORTs) or follicle-to-oocytes indexes (FOIs) (17), suggesting that a different percentage of follicles reaches maturity with a similar FSH dose as a feature linked to individual genetic background. Most of the studies focused on SNPs of the follicle-stimulating hormone (FSH) receptor gene (*FSHR*) (19–22), although the combination of different SNPs may lead to specific fertility phenotypes (23–25). However, data concerning the clinical application of pharmacogenomics in ART remain limited. Many of the previous studies aiming to elucidate the impact of gonadotropins and their receptor SNPs on OS outcome were observational and retrospective. Therefore, they were more susceptible to bias owing to the absence of a standardized OS protocol (16, 24).

The aim of this study is to evaluate the impact of gonadotropin genetics on the *in-vitro* fertilization (IVF) outcomes in infertile women treated with 150 IU FSH daily, undergoing their first OS cycle, and expected to be normo-responders, according to standardized computerized AFC.

## Method

### Study design

A multicenter, longitudinal, prospective, interventional, non-pharmacological cohort study was carried out. Women undergoing their first IVF cycle with normal ovarian reserve and attending four Italian ART centers, were enrolled.

Inclusion criteria were (i) Caucasian women, (ii) age between 34 and 39 years, (iii) body mass index (BMI) within 18 and 27 kg/m^2^, (iv) normal hypothalamic-pituitary-gonadal function, (v) basal serum FSH levels below 8 IU/L, (vi) normal ovarian reserve, established as AFC between 8 and 16, (vii) first OS cycle, and (viii) indication to either IVF or intracytoplasmic sperm injection (ICSI).

Exclusion criteria were (i) patients with ovarian cysts > 12 mm found at the first OS day, (ii) polycystic ovary syndrome (PCOS) determined by Rotterdam criteria (26), (iii) poor ovarian response according to ESHRE criteria, and (iv) endometriosis at stage III-IV of the American Society for Reproductive Medicine (ASRM) revised classification (27).

The study protocol consisted of four consecutive steps. In the first screening phase, the signature of the informed consent was collected. The first visit (V0) was performed at the time of enrollment, and anamnesis and a check for eligibility criteria were recorded. Patients screened but not eligible or not willing to participate were recorded on a separate document. The second phase represents the OS, consisting of three different visits. Visit 1 (V1) was scheduled after 2–3 days after the menstrual cycle, during which two blood samples were obtained; one was for hormone measurement, while the other one was conserved with ethylenediaminetetraacetic acid (EDTA) and sent at room temperature to the central laboratory (Unit of Endocrinology, Modena, Italy) for DNA analysis. Moreover, ultrasound evaluation was performed to calculate basal AFC. At V1, enrolled women started the treatment with 150 IU of recombinant FSH daily (Gonal-F^®^; Merck KGaA, Darmstadt, Germany). Visit 2 (V2) was performed after 6 days of FSH treatment, during which AFC and hormone assays were performed. At V2, women started with gonadotropin-releasing hormone (GnRH) antagonist (Cetrotide^®^, Merck KGaA). Visit 3 (V3) was performed when at least two follicles larger than 16 mm were detected by ultrasound. During V3, women were treated with 10,000 IU of human chorionic gonadotropin (hCG; Gonasi HP 10.000, IBSA Farmaceutici Italia S.r.l.). The trigger was induced using a GnRH agonist (Fertipeptil 0.2 mg/ml, Ferring Pharmaceuticals, Saint-Prex, Switzerland) to decrease ovarian hyperstimulation syndrome (OHSS) (28, 29) risk. The oocyte pick-ups were performed 36h after the triggering of oocyte maturation. The third study phase consisted of a visit (visit 4 – V4) in which the fresh embryo transfer was performed. In the case of OHSS risk and/or progesterone rise (≥ 2 ng/ml) on the day of hCG administration, all embryos were cryopreserved. Pregnancy was confirmed by determining serum hCGβ concentration 14 days after embryo transfer in all patients. When the pregnancy tested positive, a second test was performed 2 days later. The fourth study phase consisted of the follow-up. This was a medication-free phase in which pregnancy was followed, according to the clinical practice of each center, to evaluate chromosomal abnormalities, early preeclampsia, and the weight of the newborn. The visit 5 (V5) was performed at the end of the first trimester. The study has been approved by the ethics committee of each participating Center [*GENACOS*. *Version 2.0–15 Jun 2018 (included Amendment n.1*)]. The informed and written consent form was signed by all participants.

For ultrasound evaluation, AFC and AVC were performed using E8 expert HD live with sonoAVC, v-SRI, crossXbeam, HDflow, VCI with omiview +, and a high-resolution 4D transvaginal probe. All the clinical centers were trained by the scientific expert in AFC assessment with AVC according to the same standard parameters in order to standardize the method. A working instruction document about this procedure was delivered to each center.

### Study endpoints

The primary endpoint of the study was the number of retrieved oocytes. The primary endpoint was evaluated considering the allelic state of the c.-29G>A *FSHR* SNP (rs1394205).

Secondary outcomes were (v) duration of OS indicated as days of stimulation; (vi) number of mature oocytes; (vii) number of fertilized 2PN oocytes; (viii) number of embryos developed, transferred, and cryopreserved; (ix) implantation rate; (x) pregnancy rate per started cycle; (xi) pregnancy rate per embryo transfer; and (xii) cumulative ongoing pregnancy rate per started cycle. Ovarian sensitivity was also evaluated using follicular output rate (FORT) and follicle-to-oocytes indexes (FOI) (17).

All these parameters were evaluated in association with the following SNPs: *FSHR* c.-29G>A (rs1394205); *FSHR* c.2039A>G, p.N680S (rs6166); *FSHB* c.-211G>T, (rs10835638); *LHCGR* c.942G>A, p.S312N (rs2293275); *LHCGR* c.872A>G, p.N291S (rs12470652); LHβ genetic variant c.82T>C, p.W8R (rs1800447) and c.104T>C, p.I15T (rs34349826). These SNPs were selected considering literature evidence that showed an association of these genetic variants to ovarian response and IVF outcome (22, 30–32).

### Genotype analysis

All DNA analyses were performed after the OS, at the Unit of Endocrinology, Department of Biomedical, Metabolic and Neural Sciences (University of Modena and Reggio Emilia, Modena, Italy). Genomic DNA was extracted from white blood cells using the automated extractor EZ1 Advanced XL (Qiagen, Hilden, Germany) and quantified by a NanoDrop™ 2000 spectrophotometer (Thermo Fisher Scientific, Waltham, MA, USA).

The analysis of *FSHR* c.-29G>A, *FSHR* c.2039A>G leading to the amino acid change p.N680S at the protein level, as well as of *FSHB* c.-211G>T SNPs, was performed by high-resolution melting (HRM) technology on a CFX96 Real-time PCR detection system (Bio-Rad Laboratories Inc., Hercules, CA, USA), using the following primer pairs: forward (Fw) 5’-ATAATTATGCATCCATCCAC-3’ and reverse (Rev) 5’-GAGATCTGTGGAGGTTTT-3’ for *FSHR* c.-29G>A; Fw 5’-AACACCCATCCAAGGAAT-3’ and Rev 5’-ATGACTTAGAGGGACAAG-3’ for *FSHR* c.2039A>G; Fw 5’-GGTGTGCTACTGTATCAA-3’ and Rev 5’-AAAGTAGTCTAAACGCAGTA-3’ for *FSHB* SNP c.-211G>T.

Genotyping of LHCGR p.S312N and p.N291S was performed by Sanger sequencing analysis, as previously described (33, 34) using the following primer pairs: Fw 5’-AGGCCAATGTGGAAAGGAGAG-3’ and Rev 5’-TGCAACAGCTCCGTAACCAA-3’. The analysis of the LHβ V-LH variant (p.W8R and p.I15T) was performed as previously described (35). Briefly, the cDNA region containing the *LHB* variants was amplified by PCR, using the primer pair Fw 5’- GAAGCAGTGTCCTTGTCCCA-3’ and Rev 5’- GAAGAGGAGGCCTGAGAGTT-3’, and resulting in a 622 bp fragment. For each sample, PCR products corresponding to p.W8R and p.I15T SNPs were digested by the *Nco*I and the *Bse*GI restriction enzymes (Thermo Scientific, US), respectively. The genotype was determined by the specific restriction fragments pattern observed by 3% agarose gel migration.

### Determination of sample size

Many studies evaluated *FSHR* gene promoter *in-vitro* activity (position c.-29G>A; rs1394205), revealing decreased transcriptional activity of 56 ± 8% than that of the G allele. These data were corroborated by a study in 100 women attending ART, where authors found reduced relative *FSHR* mRNA transcripts linked to *FSHR* c.-29G>A A homozygosity (36). In the dominant model, relative *FSHR* mRNA expression was of 0.12 for G/G allele, 0.07 for G/A, and 0.02 for A/A (36). Thus, power analysis was performed (by G*Power software, version 3.1.9.2) assuming a variation of 0.075 of relative *FSHR* mRNA expression produced by two genotypes. Differences between two independent means were considered, α error probability was set to 0.05 and allocation ratio to 0.89, considering previous observational study (23, 36). According to the statistical power of 80%, sample size was estimated in 184 patients. Considering a dropout rate of 10%, the total number was of at least 202 patients.

### Statistical methods analysis

The Kolmogorov–Smirnov test was used for evaluation of the parameters’ distribution. Mann–Whitney’s *U* test and Kruskal–Wallis test were used for comparison of not-normally distributed variables, whereas t-test and ANOVA univariate analyses were used for normally distributed ones. Pregnancy outcomes were evaluated using a logistic regression model adjusting for age, day of the transfer, and number of transferred embryos. Statistical analysis was performed using the “Statistical Package for the Social Sciences” software for Macintosh (version 20.0; SPSS Inc., Chicago, IL). *P*-values < 0.05 were considered as statistically significant.

Genotypic association tests were performed considering each SNP alone, assuming codominant, dominant, recessive, overdominant, or log-additive genetic models. Moreover, the haplotype obtained by SNP combination was generated and its impact on the endpoint was evaluated by multivariate analyses. Genotypic association analyses were performed using SNPstats. Linkage disequilibrium was evaluated using SNPStat.

## Results

A total of 119 patients were initially enrolled in the study. Eight patients withdrew from the study immediately after providing informed consent, before beginning OS. Five more patients dropped out after commencing OS. Finally, 106 women completed the OS protocol and underwent oocyte retrieval. Baseline characteristics of the participants are detailed in **Table 1**. Considering the difficulties in the recruitment process due to relatively strict inclusion criteria and the lack of significant findings regarding the primary outcome (number of oocytes retrieved), the enrollment was interrupted. This decision was carried out considering an internal *post-hoc* analysis of our data. Considering these issues, a *post hoc* analysis was performed, and, setting the effect size to 0.165, the statistical power was 71.7%.

**Table 1:** Patients baseline characteristics.

Patients characteristics	Results
Number of patients achieving pick up	106
Age (years)	36.3 (1.7)
Weight (kg)	59.0 (7.7)
Height (cm)	164 (6)
BMI (kg/m^2^)	21.9 (2.5)
Smoking status	
Current smoker	30 (28.3)
Former smoker	1 (0.9)
Non smoker	83 (70.9)
Infertility duration (years)	3.6 (2.2)
Causes of infertility	
Only female	18 (15.4)
Only male	51 (43.6)
Male and female	1 (0.8)
Unexplained	47 (40.2)
Tubaric factor (*n*)	15 (12.8)
FSH IU/L	6.1 (1.7)
LH IU/L	4.1 (1.6)
AMH ng/ml	2.4 (1.0)
E2 pg/ml	48.4 (53.4)

Data are presented as mean (SD) for continuous data and number (percentage) for categorical ones.

Embryo transfer was performed in 76.2% of cycles, equally distributed between day 3 (36.2%) and day 5 (33.3%). Only 6.7% of embryo transfers were performed at day 2. In 21% of cycles started, all embryos were frozen due to fresh embryo transfer contraindications. OS outcomes are reported in **Table 2**.

**Table 2:** Stimulation cycle outcomes.

Stimulation cycle characteristics	Results
Number of patients	106
Follicle number at Visit 1	13.4 (2.5)
Ovulation induction ◼ HCG ◼ a-GnRH	95 (89.6) 11 (10.4)
Number of follicles ≥ 16mm at induction phase	5.8 (2.5)
Number of retrieved oocytes at pick-up	8.4 (4.2)
Mature oocytes (%)	85.7 (64.0–100)
Number of fertilized oocytes	6.0 (3.2)
Fertilized oocytes (%)	75.0 (60.1–88.2)
Cycles without fertilized oocytes	1 (0.9)
Cycles without developed embryos	3 (2.9)
Cycles with fresh embryo transfer (ET) ◼ Day 2 ◼ Day 3 ◼ Day 5	80 (76.2) 7 (6.7) 38 (36.2) 35 (33.3)
“Freeze all” cycles Due to OHSS risk Due to high progesterone levels Other causes	22 (21.0) 12 (54.6) 7 (31.8) 3 (13.6)
Number of embryos obtained	2.8 (1.7)
Positive β-hCG, per fresh ET	49.4
Ongoing pregnancies, fresh ET	37.8
Cumulative positive β-Hcg, per pick-up	53.8
Cumulative ongoing pregnancies per pick-up	36.8

Data are presented as mean (SD) for continuous data and number (percentage) for categorical ones.

Seven different SNPs have been analyzed for patients recruited. All SNPs were in “Hardy-Weinberg equilibrium” except for LHCGR p.N291S (**Table 3**). SNPs with an allele frequency lower than 10%, or not in Hardy-Weinberg equilibrium, were removed from further analysis, and OS characteristics were compared considering the remaining five SNPs (*FSHR* c.-29G>A, FSHR p.N680S, *FSHB* c.-211G>T, LHCGR p.S312N, and LHβ V-LH p.W8R). One patient was *FSHB* c.-211 G>T T homozygous and achieved oocyte pick-up.

**Table 3:** Patients’ genotype.

Name	ID (Reference minor allele frequency)	Allele1/ allele2	Frequency allele 1	Frequency allele 2	Homo- zygous allele 1	Hetero-zygous	Homo- zygous allele 2	Hardy Weinberg equilibrium
*FSHR* c.-29G/A	rs1394205 (A 28.9)	G/A	70.9	29.1	59 (50.4)	48 (41.0)	10 (8.6)	yes
*FSHR* p.N680S	rs6166 (G 44.9)	A/G	53.8	46.2	36 (30.89	54 (46.1)	27 (23.1)	yes
*FSHB* c.-211 G > T	rs10835638 (T 14.3)	G/T	85.5	14.5	85 (72.7)	30 (25.6)	2 (1.7)	yes
*LHB* p.W8R	rs1800447 (G 9.5)	A/G	92.3	7.7	99 (84.6)	18 (15.4)	0 (0.0)	yes
LHB p.I15T	rs34349826 (G 8.9)	A/G	92.3	7.7	99 (84.6)	18 (15.4)	0 (0.0)	yes
*LHCGR* p.N291S	rs12470652 (C 4.6)	T/C	96.6	3.4	110 (94.0)	6 (5.1)	1 (0.9)	no
*LHCGR* p.S312N	rs2293275 (A 36.0)	G/A	63.7	36.3	49 (41.9)	51 (43.6)	17 (14.5)	yes

Allele 1, major allele. Values are expressed in percent or numbers (percentage).

The reference minor allele frequency (percentage) is referred to the aggregate “European” population indexed in the 1000 Genomes database, and accessed through the National Center for Biotechnology Information SNP database (https://www.ncbi.nlm.nih.gov/snp).

### Number of oocytes retrieved; metaphase II oocytes; 2PN oocytes

The total number of oocytes retrieved did not change considering the genotype in the codominant, dominant, recessive, overdominant, or log-additive genetic models (**Table 4**). Similarly, multivariate analyses did not detect any specific association between haplotype and number of oocytes retrieved (**Table 5**).

**Table 4:** Genotypic association tests performed considering each SNP alone for the number of retrieved oocytes.

Model	Genotype	*n*	Response mean (SE)	Difference (95% CI)	*P*-value
*FSHR* c.-29G > A					
Codominant	GG	59	7.64 (0.61)	0.00	0.380
	GA	48	7.12 (0.70)	−0.52 (−2.31 to 1.27)	
	AA	10	9.4 (1.25)	1.76 (−1.39 to 4.90)	
Dominant	GG	59	7.64 (0.61)	0.00	0.880
	GA-AA	58	7.52 (0.62)	−0.13 (−1.83 to 1.58)	
Recessive	GG-GA	107	7.41 (0.46)	0.00	0.200
	AA	10	9.4 (1.25)	1.99 (−1.04 to 5.02)	
Overdominant	GG-AA	69	7.9 (0.55)	0.00	0.380
	GA	48	7.12 (0.7)	−0.77 (−2.50 to 0.95)	
FSHR p.N680S					
Codominant	NN	36	7.42 (0.77)	0.00	0.970
	NS	54	7.67 (0.59)	0.25 (−1.74 to 2.24)	
	SS	27	7.63 (1.06)	0.21 (−2.15 to 2.57)	
Dominant	NN	36	7.42 (0.77)	0.00	0.800
	NS-SS	81	7.65 (0.53)	0.24 (−1.61 to 2.09)	
Recessive	NN-NS	90	7.57 (0.47)	0.00	0.950
	SS	27	7.63 (1.06)	0.06 (−1.96 to 2.09)	
Overdominant	NN-SS	63	7.51 (0.63)	0.00	0.860
	NS	54	7.67 (0.59)	0.16 (−1.55 to 1.87)	
FSHB c.-211G > T					
Codominant	GG	85	7.52 (0.49)	0.00	0.900
	GT	30	7.67 (0.88)	0.15 (−1.82 to 2.12)	
	TT	2	9 (9)	1.48 (−5.14 to 8.11)	
Dominant	GG	85	7.52 (0.49)	0.00	0.810
	GT-TT	32	7.75 (0.92)	0.23 (−1.68 to 2.15)	
Recessive	GG-GT	115	7.56 (0.43)	0.00	0.670
	TT	2	9 (9)	1.44 (−5.13 to 8.02)	
Overdominant	GG-TT	87	7.55 (0.5)	0.00	0.910
	GT	30	7.67 (0.88)	0.11 (−1.84 to 2.07)	
LHB V-LH p.W8R					
Codominant	WW	99	7.34 (0.48)	0.00	0.200
	WR	18	8.89 (1)	1.55 (−0.80 to 3.89)	
LHCGR p.S312N					
Codominant	CC	49	7.51 (0.67)	0.00	0.900
	TC	51	7.49 (0.7)	−0.02 (−1.87 to 1.83)	
	TT	17	8.06 (0.91)	0.55 (−2.06 to 3.15)	
Dominant	CC	49	7.51 (0.67)	0.00	0.890
	TC-TT	68	7.63 (0.57)	0.12 (−1.61 to 1.85)	
Recessive	CC-TC	100	7.5 (0.48)	0.00	0.650
	TT	17	8.06 (0.91)	0.56 (−1.86 to 2.98)	
Overdominant	CC-TT	66	7.65 (0.55)	0.00	0.850
	TC	51	7.49 (0.7)	−0.16 (−1.88 to 1.56)	

**Table 5:** Multivariate analysis performed considering the ovarian stimulation parameters as dependent variables, and the allele combination as independent ones.

haplotype n	*FSHR* c.-29G>A	FSHR c.2039A>G (p.N680S)	*FSHB* c.-211G>T	V-LHβ c.82T>C (p.W8R)	LHCGR c.942G>A (p.S312N)	Frequency	Difference (95% CI)	*P-*value
Oocytes retrieved								
1	G	N	G	W	S	0.2235	0.00	—
2	G	S	G	W	S	0.1825	0.82 (−1.2 to 2.83)	0.430
3	G	S	G	W	N	0.0997	1.31 (−0.92 to 3.54)	0.250
4	A	N	G	W	S	0.0937	1.87 (−0.88 to 4.62)	0.180
5	G	N	G	W	N	0.0759	2.19 (−0.95 to 5.34)	0.170
6	A	N	G	W	N	0.0687	0.77 (−2.57 to 4.11)	0.650
7	A	S	G	W	N	0.0613	−2.31 (−5.38 to 0.75)	0.140
8	G	S	T	W	S	0.0521	−0.54 (−3.49 to 2.41)	0.720
9	A	S	G	W	S	0.0494	0.82 (−2.02 to 3.66)	0.570
10	G	N	T	W	N	0.0411	−0.72 (−4.14 to 2.7)	0.680
Mature oocytes								
1	G	N	G	W	S	0.2299	0.00	—
2	G	S	G	W	S	0.1415	0.82 (−1.01 to 2.65)	0.380
3	A	N	G	W	S	0.1134	0.51 (−1.45 to 2.47)	0.610
4	G	S	G	W	N	0.1108	−0.25 (−2.28 to 1.79)	0.810
5	G	N	G	W	N	0.066	1.33 (−0.84 to 3.51)	0.230
6	A	N	G	W	N	0.0564	1.63 (−0.75 to 4.01)	0.180
**7**	**A**	**S**	**G**	**W**	**S**	**0.0481**	**2.26 (0.04–4.47)**	**0.048**
8	G	S	T	W	S	0.0445	−0.03 (−2.55 to 2.49)	0.980
9	A	S	G	W	N	0.0276	−2.54 (−6.18 to 1.1)	0.170
10	G	N	T	W	N	0.0223	3.56 (−0.01 to 7.12)	0.052
FOI								
1	G	N	G	W	S	0.2299	0.00	—
**2**	**G**	**S**	**G**	**W**	**S**	**0.1376**	**0.18 (0.04–0.33)**	**0.011**
3	A	N	G	W	S	0.1133	0.13 (−0.03 to 0.28)	0.100
4	G	S	G	W	N	0.1042	0.02 (−0.13 to 0.18)	0.780
**5**	**G**	**N**	**G**	**W**	**N**	**0.0736**	**0.18 (0.01–0.34)**	**0.037**
6	A	N	G	W	N	0.0508	0.13 (−0.08 to 0.35)	0.220
7	G	S	T	W	S	0.0486	0.11 (−0.09 to 0.31)	0.280
8	A	S	G	W	S	0.0471	−0.01 (−0.19 to 0.17)	0.940
9	A	S	G	W	N	0.0308	−0.19 (−0.44 to 0.05)	0.120
10	G	S	G	R	S	0.0249	0.2 (−0.05 to 0.46)	0.120
Cumulative FSH dose								
1	G	N	G	W	S	0.2249	0.00	–
**2**	G	S	G	W	S	0.1444	5.5 (−47.01 to 58.01)	0.840
**3**	**G**	**S**	**G**	**W**	**N**	**0.1055**	**119.82 (53.45–186.19)**	**< 0.001**
4	A	N	G	W	S	0.0996	−22.77 (−75.91 to 30.37)	0.400
**5**	**G**	**N**	**G**	**W**	**N**	**0.0733**	**150.03 (88.61–211.45)**	**< 0.001**
**6**	**A**	**N**	**G**	**W**	**N**	**0.0535**	**82.90 (60.60–105.20)**	**< 0.001**
**7**	**A**	**S**	**G**	**W**	**S**	**0.0527**	**113.01 (92.29–133.74)**	**< 0.001**
**8**	**G**	**N**	**T**	**W**	**S**	**0.0334**	**206.51 (195.29–217.72)**	**< 0.001**
**9**	**G**	**S**	**T**	**W**	**S**	**0.0325**	**−22.21 (−27.84** to **−16.59)**	**< 0.001**
**10**	**A**	**S**	**G**	**W**	**N**	**0.0309**	**−104.09 (−109.19** to **−98.99)**	**< 0.001**
Ovarian stimulation duration								
1	G	N	G	W	S	0.2144	0.00	—
2	G	S	G	W	S	0.1471	0.33 (−0.21 to 0.87)	0.230
3	A	N	G	W	S	0.1052	0.14 (−0.49 to 0.78)	0.660
4	G	S	G	W	N	0.0947	0.18 (−0.44 to 0.8)	0.570
**5**	**G**	**N**	**G**	**W**	**N**	**0.0748**	**1.34 (0.76–1.93)**	**< 0.001**
6	A	N	G	W	N	0.0611	0.04 (−0.73 to 0.8)	0.930
7	A	S	G	W	**S**	0.0443	0.6 (−0.13 to 1.33)	0.110
8	G	S	T	W	**S**	0.0402	0.09 (−0.71 to 0.89)	0.830
**9**	**G**	**N**	**T**	**W**	**S**	**0.0360**	**1.74 (0.74–2.74)**	**< 0.001**
**10**	**A**	**S**	**G**	**W**	**N**	**0.0340**	**1.33 (0.4–2.25)**	**0.005**
Follicle output rate (FORT)								
1	G	N	G	W	S	0.2445	0.00	–
2	G	S	G	W	S	0.1337	0.16 (−0.03 to 0.35)	0.096
**3**	**A**	**N**	**G**	**W**	**S**	**0.1038**	**0.26 (0.05–0.47)**	**0.016**
4	G	S	G	W	N	0.0937	−0.02 (−0.25 to 0.21)	0.850
**5**	**G**	**N**	**G**	**W**	**N**	**0.0632**	**0.30 (0.06–0.54)**	**0.015**
6	G	S	T	W	S	0.0528	−0.03 (−0.29 to 0.23)	0.810
7	A	S	G	W	N	0.0515	−0.12 (−0.39 to 0.16)	0.410
8	A	S	G	W	S	0.0496	−0.11 (−0.35 to 0.14)	0.380
9	A	N	G	W	N	0.0459	0.27 (−0.02 to 0.55)	0.070
10	G	N	T	W	N	0.0354	−0.21 (−0.53 to 0.10)	0.190
2PN oocytes								
1	G	N	G	W	C	0.2442	0.00	—
2	G	S	G	W	C	0.1355	−0.01 (−1.85 to 1.83)	0.990
3	A	N	G	W	C	0.1134	0.41 (−1.33 to 2.15)	0.640
4	G	S	G	W	T	0.1042	0.27 (−.58 to 2.12)	0.770
5	G	N	G	W	T	0.0653	0.35 (−2.1 to 2.8)	0.780
6	G	S	T	W	C	0.0473	0.77 (−1.42 to 2.96)	0.490
7	A	N	G	W	T	0.0464	0.89 (−1.54 to 3.32)	0.470
8	A	S	G	W	C	0.0426	1.52 (−0.61 to 3.65)	0.160
9	A	S	G	W	T	0.0406	−2.67 (−5.42 to 0.08)	0.059
10	G	S	G	R	C	0.0269	−0.93 (−3.67 to 1.82)	0.510

Only the ten most frequent allele combinations are shown.

p<0.05 are indicated with bold.

A significantly higher number of mature oocytes was retrieved when T homozygous (FSHB c.-211G>T) was expressed in codominant and recessive models (**Table 6**). Multivariate analysis confirmed the impact of haplotype on mature oocytes retrieved (**Table 5**).

**Table 6:** Genotypic association tests performed considering each SNP alone for the number of mature oocytes retrieved.

Model	Genotype	*n*	Response mean (SE)	Difference (95% CI)	*P*-value
*FSHR* c.-29G>A					
**Codominant**	GG	53	6.04 (0.45)	0.00	0.430
	GA	43	6.16 (0.54)	0.13 (−1.28 to 1.53)	
	AA	10	7.6 (1.35)	1.56 (−0.80 to 3.92)	
**Dominant**	GG	53	6.04 (0.45)	0.00	0.560
	GA-AA	53	6.43 (0.51)	0.40 (−0.94 to 1.73)	
**Recessive**	GG-GA	96	6.09 (0.35)	0.00	0.200
	AA	10	7.6 (1.35)	1.51 (−0.76 to 3.77)	
**Overdominant**	GG-AA	63	6.29 (0.44)	0.00	0.860
	GA	43	6.16 (0.54)	−0.12 (−1.48 to 1.24)	
FSHR p.N680S					
**Codominant**	NN	32	6.44 (0.62)	0.00	0.820
	NS	52	6.02 (0.51)	−0.42 (−1.97 to 1.13)	
	SS	22	6.45 (0.69)	0.02 (−1.89 to 1.93)	
**Dominant**	NN	32	6.44 (0.62)	0.00	0.700
	NS-SS	74	6.15 (0.41)	−0.29 (−1.74 to 1.16)	
**Recessive**	NN-NS	84	6.18 (0.39)	0.00	0.740
	SS	22	6.45 (0.69)	0.28 (−1.37 to 1.92)	
**Overdominant**	NN-SS	54	6.44 (0.46)	0.00	0.530
	NS	52	6.02 (0.51)	−0.43 (−1.76 to 0.91)	
FSHβ c.-211G>T					
**Codominant**	GG	77	6.05 (0.39)	0.00	**0.035**
	GT	28	6.43 (0.64)	0.38 (−1.10 to 1.85)	
	TT	1	15 (0)	**8.95 (2.22–15.68)**	
**Dominant**	GG	77	6.05 (0.39)	0.00	0.380
	GT-TT	29	6.72 (0.69)	0.67 (−0.82 to 2.16)	
**Recessive**	GG-GT	105	6.15 (0.33)	0.00	**0.011**
	TT	1	15 (0)	**8.85 (2.15–15.54)**	
**Overdominant**	GG-TT	78	6.17 (0.4)	0.00	0.740
	GT	28	6.43 (0.64)	0.26 (−1.25 to 1.78)	
LHβ V-LH p.W8R					
**Codominant**	WW	88	6.4 (0.39)	0.00	0.180
	WR	18	5.44 (0.63)	−0.95 (−2.72 to 0.82)	
LHCGR p.S312N					
**Codominant**	SS	45	6.13 (0.58)	0.00	0.960
	NS	45	6.29 (0.5)	0.16 (−1.30 to 1.61)	
	NN	16	6.38 (0.73)	0.24 (−1.77 to 2.25)	
**Dominant**	SS	45	6.13 (0.58)	0.00	0.800
	NS-NN	61	6.31 (0.41)	0.18 (−1.17 to 1.53)	
**Recessive**	SS-NS	90	6.21 (0.38)	0.00	0.860
	NN	16	6.38 (0.73)	0.16 (−1.70 to 2.03)	
**Overdominant**	SS-NN	61	6.2 (0.46)	0.00	0.890
	SS	45	6.29 (0.5)	0.09 (−1.26 to 1.44)	

p<0.05 are indicated with bold.

Similarly, 2PN oocytes were significantly higher when the FSHB c.-211G>T T allele was present, both in codominant and recessive models (**Table 7**). However, no specific SNPs or haplotypes able to describe 2PN variation were detected by multivariate analysis (**Table 5**). Considering that only a very small number of women expressed allele T, these findings might be fortuitous.

**Table 7:** Genotypic association tests performed considering each SNP alone for the number of 2PN oocytes retrieved.

Model	Genotype	*n*	Response mean (SE)	Difference (95% CI)	*P*-value
*FSHR* c.-29G>A					
**Codominant**	GG	52	6.04 (0.43)	0.00	0.230
	GA	43	5.67 (0.5)	−0.36 (−1.65 to 0.92)	
	AA	10	7.6 (1)	1.56 (−0.59 to 3.72)	
**Dominant**	GG	52	6.04 (0.43)	0.00	0.999
	GA-AA	53	6.04 (0.46)	−0.00 (−1.23 to 1.23)	
**Recessive**	GG-GA	95	5.87 (0.33)	0.00	0.100
	AA	10	7.6 (1)	1.73 (−0.34 to 3.79)	
**Overdominant**	GG-AA	62	6.29 (0.4)	0.00	0.330
	GA	43	5.67 (0.5)	−0.62 (−1.86 to 0.63)	
FSHR p.N680S					
**Codominant**	NN	31	6.23 (0.57)	0.00	0.510
	NS	52	5.69 (0.46)	−0.53 (−1.96 to 0.89)	
	SS	22	6.59 (0.65)	0.37 (−1.39 to 2.12)	
**Dominant**	NN	31	6.23 (0.57)	0.00	0.700
	NS-SS	74	5.96 (0.37)	−0.27 (−1.61 to 1.08)	
**Recessive**	NN-NS	83	5.89 (0.36)	0.00	0.360
	SS	22	6.59 (0.65)	0.70 (−0.81 to 2.20)	
**Overdominant**	NN-SS	53	6.38 (0.43)	0.00	0.270
	NS	52	5.69 (0.46)	−0.69 (−1.91 to 0.54)	
*FSHB* c.-211G>T					
**Codominant**	GG	76	5.86 (0.33)	0.00	**0.036**
	GT	28	6.25 (0.69)	0.39 (−0.96 to 1.75)	
	TT	1	14 (0)	**8.14 (1.98–14.31)**	
**Dominant**	GG	76	5.86 (0.33)	0.00	0.350
	GT-TT	29	6.52 (0.72)	0.66 (−0.71 to 2.03)	
**Recessive**	GG-GT	104	5.96 (0.31)	0.00	**0.012**
	TT	1	14 (0)	**8.04 (1.90–14.17)**	
**Overdominant**	GG-TT	77	5.96 (0.35)	0.00	0.680
	GT	28	6.25 (0.69)	0.29 (−1.10 to 1.68)	
LHβ V-LH p.W8R					
**Codominant**	WW	88	6.07 (0.35)	0.00	0.830
	WR	17	5.88 (0.66)	−0.19 (−1.85 to 1.48)	
LHCGR p.S312N					
**Codominant**	SS	44	5.89 (0.52)	0.00	0.900
	NS	45	6.2 (0.5)	0.31 (−1.03 to 1.65)	
	NN	16	6 (0.56)	0.11 (−1.73 to 1.96)	
**Dominant**	SS	44	5.89 (0.52)	0.00	0.680
	NS-NN	61	6.15 (0.39)	0.26 (−0.98 to 1.51)	
**Recessive**	SS-NS	89	6.04 (0.36)	0.00	0.960
	NN	16	6 (0.56)	−0.04 (−1.76 to 1.67)	
**Overdominant**	SS-NN	60	5.92 (0.4)	0.00	0.660
	SS	45	6.2 (0.5)	0.28 (−0.96 to 1.52)	

p<0.05 are indicated with bold.

### Ovarian sensitivity indexes: FOI and FORT

FOI was significantly influenced by FSHB c.-211G>T (**Table 8**). In particular, FOI was higher in FSHB c.-211G>T T homozygous patients, both in codominant and recessive models (**Table 8**). Even in this circumstance the rarity of allele T should be taken into account in the interpretation of data.

**Table 8:** Genotypic association tests performed considering each SNP alone for the follicle on oocytes index (FOI).

Model	Genotype	*n*	Response mean (SE)	Difference (95% CI)	*P*-value
*FSHR* c.-29G>A					
**Codominant**	GG	53	0.65 (0.04)	0.00	0.850
	GA	42	0.61 (0.05)	−0.03 (−0.15 to 0.09)	
	AA	10	0.63 (0.08)	−0.02 (−0.22 to 0.18)	
**Dominant**	GG	53	0.65 (0.04)	0.00	0.590
	GA-AA	52	0.62 (0.04)	−0.03 (−0.14 to 0.08)	
**Recessive**	GG-GA	95	0.63 (0.03)	0.00	0.990
	AA	10	0.63 (0.08)	−0.00 (−0.19 to 0.19)	
**Overdominant**	GG-AA	63	0.65 (0.04)	0.00	0.590
	GA	42	0.61 (0.05)	−0.03 (−0.15 to 0.08)	
FSHR p.N680S					
**Codominant**	NN	32	0.61 (0.04)	0.00	0.510
	NS	51	0.62 (0.04)	0.01 (−0.12 to 0.14)	
	SS	22	0.7 (0.07)	0.09 (−0.07 to 0.25)	
**Dominant**	NN	32	0.61 (0.04)	0.00	0.610
	NS-SS	73	0.64 (0.04)	0.03 (−0.09 to 0.15)	
**Recessive**	NN-NS	83	0.62 (0.03)	0.00	0.240
	SS	22	0.7 (0.07)	0.08 (−0.06 to 0.22)	
**Overdominant**	NN-SS	54	0.65 (0.04)	0.00	0.630
	NS	51	0.62 (0.04)	−0.03 (−0.14 to 0.09)	
*FSHB* c.-211G>T					
**Codominant**	GG	76	0.64 (0.03)	0.00	**0.027**
	GT	28	0.59 (0.06)	−0.04 (−0.17 to 0.08)	
	TT	1	1.38 (0)	**0.75 (0.18–1.31)**	
**Dominant**	GG	76	0.64 (0.03)	0.00	0.810
	GT-TT	29	0.62 (0.06)	−0.02 (−0.14 to 0.11)	
**Recessive**	GG-GT	104	0.63 (0.03)	0.00	**0.009**
	TT	1	1.38 (0)	**0.76 (0.20** to **1.32)**	
**Overdominant**	GG-TT	77	0.65 (0.03)	0.00	0.420
	GT	28	0.59 (0.06)	−0.05 (−0.18 to 0.07)	
LHβ V-LH p.W8R					
**Codominant**	WW	87	0.62 (0.03)	0.00	0.180
	WR	18	0.72 (0.07)	0.10 (−0.05 to 0.25)	
**LHCGR p.S312N**					
**Codominant**	SS	45	0.64 (0.05)	0.00	0.990
	NS	44	0.63 (0.04)	−0.01 (−0.13 to 0.11)	
	NN	16	0.63 (0.06)	−0.01 (−0.18 to 0.16)	
**Dominant**	SS	45	0.64 (0.05)	0.00	0.880
	NS-NN	60	0.63 (0.03)	−0.01 (−0.12 to 0.10)	
**Recessive**	SS-NS	89	0.63 (0.03)	0.00	0.920
	NN	16	0.63 (0.06)	−0.01 (−0.17 to 0.15)	
**Overdominant**	SS-NN	61	0.63 (0.04)	0.00	0.940
	SS	45	0.64 (0.05)	0.00	

p<0.05 are indicated with bold.

In multivariate analysis, two different haplotypes were associated with FOI (*p* = 0.005), which was higher when the S allele in both FSHR p.N680S and LHCGR p.S312N were combined (**Table 5**). Similar results were found when FOI was considered for the N allele in both FSHR p.N680S and LHCGR p.S312N combined (**Table 5**).

The FORT was not significantly different among SNP haplotypes, irrespective of the genetic model considered (**Supplementary Table S1**). However, in the multivariate analysis, significant influence of haplotypes generated by the five SNPs was detected (*p* = 0.003) in association with FORT, representing 10.3% of the entire cohort (**Table 5**).

### Days of stimulation

OS duration was influenced by three genotypes (**Table 9**). In the overdominant model, FSHR p.N680S heterozygosis showed a significantly reduced OS compared to other genotypes (**Table 9**), while *FSHB* c.-211G>T “T” and LHCGR p.N312S “S” alleles were linked to prolonged stimulation in codominant, dominant, and overdominant models (**Table 9**).

**Table 9:** Genotypic association tests performed considering each SNP alone for the duration of ovarian stimulation (OS).

Model	Genotype	*n*	Response mean (SE)	Difference (95% CI)	*P*-value
*FSHR* c.-29G>A					
**Codominant**	GG	53	7.98 (0.18)	0.00	0.560
	GA	44	8.07 (0.18)	0.09 (−0.41 to 0.58)	
	AA	10	7.60 (0.27)	−0.8 (−1.22 to 0.46)	
**Dominant**	GG	53	7.98 (0.18)	0.00	0.999
	GA-AA	54	7.98 (0.16)	0.00 (−0.47 to 0.47)	
**Recessive**	GG-GA	97	8.02 (0.13)	0.000	0.310
	AA	10	7.60 (0.27)	−0.42 (−1.23 to 0.38)	
**Overdominant**	GG-AA	63	7.92 (0.16)	0.00	0.650
	GA	44	8.07 (0.18)	0.15 (−0.33 to 0.62)	
FSHR p.N680S					
**Codominant**	NN	32	8.16 (0.21)	0.00	0.110
	NS	52	7.73 (0.17)	−0.43 (−0.96 to 0.11)	
	SS	23	8.30 (0.26)	0.15 (−0.51 to 0.80)	
**Dominant**	NN	32	8.16 (0.21)	0.00	0.340
	NS-SS	75	7.91 (0.15)	−0.25 (−0.76 to 0.26)	
**Recessive**	NN-NS	84	7.89 (0.13)	0.00	0.160
	SS	23	8.30 (0.26)	0.41 (−0.16 to 0.98)	
**Overdominant**	NN-SS	55	8.22 (0.16)	0.00	**0.041**
	NS	52	**7.73 (0.17)**	−**0.49 (−0.95 to −0.03)**	
*FSHB* c.-211G>T					
**Codominant**	GG	77	7.83 (0.14)	0.00	**0.030**
	GT	29	8.38 (0.23)	**0.55 (0.33–1.07)**	
	TT	1	8.00 (0.00)	0.17 (−2.24 to 1.05)	
**Dominant**	GG	77	7.83 (0.14)	0.00	**0.044**
	GT-TT	30	8.37 (0.23)	**0.54 (0.02–1.05)**	
**Recessive**	GG-GT	106	7.98 (0.12)	0.00	0.990
	TT	1	8.00 (0.00)	0.02 (−2.43 to 2.46)	
**Overdominant**	GG-TT	78	7.83 (0.14)	0.00	**0.042**
	GT	29	8.38 (0.23)	**0.55 (0.03–1.07)**	
LHβ V-LH p.W8R					
**Codominant**	WW	89	8.02 (0.12)	0.00	0.450
	WR	18	7.78 (0.36)	−0.24 (−0.87 to 0.38)	
LHCGR p.S312N					
**Codominant**	SS	45	7.56 (0.15)	0.00	**0.007**
	NS	46	8.24 (0.19)	**0.68 (0.19–1.17)**	
	NN	16	8.44 (0.34)	**0.88 (0.20–1.56)**	
**Dominant**	SS	45	7.56 (0.15)	0.00	**0.002**
	NS-NN	62	8.29 (0.16)	**0.73 (0.28–1.19)**	
**Recessive**	SS-NS	31	7.90 (0.13)	0.00	0.110
	NN	16	8.44 (0.34)	0.54 (−0.12 to 1.19)	
**Overdominant**	SS-NN	61	7.79 (0.15)	0.00	0.061
	SS	46	8.24 (0.19)	0.45 (−0.02 to 0.92)	

p<0.05 are indicated with bold.

Multivariate analysis confirmed the impact of haplotypes generated by the five SNPs (*p* = 0.009), revealing that the combination of *FSHR* c.-29G>A G allele, FSHR p.N680S N allele, *FSHB* c.-211G>T G allele, LHβ V-LH p.W8R W allele, and LHβ and LHCGR p.S312N N allele was associated with longer stimulation (**Table 5**). Overall, this combination was expressed in 7.4% of the study population.

### Number of embryos developed, transferred and cryopreserved

No significant difference was observed among SNP haplotypes (**Supplementary Table S2**) in both separate and multivariate analyses, irrespective to the genetic model considered.

### Implantation rate; pregnancy rate per started cycle; pregnancy rate per embryo transfer; cumulative ongoing pregnancy rate per started cycle

No significant difference was observed among SNP haplotypes, irrespective to the genetic model considered. Given the limited sample size, multivariate analysis was not carried out.

## Discussion

Our study evaluated prospectively the potential role of five SNPs of gonadotropin and their receptor genes on the response to OS in normo-responder women undergoing ART. Our analysis does not clearly identify a genetic haplotype associated with the number of oocytes retrieved after standard antagonist COS protocol. However, SNPs or combinations of them could impact OS parameters. In particular, FSHR p.N680S and LHCGR p.N312S SNPs influence the duration of the stimulation per se. Concerning the LHβ V-LH variant (p.W8R and p.I15T), we did not detect any relevant findings, likely due to reduced sample size and ethnicity-related issues, opposite to what was previously demonstrated (37). Moreover, although the evidence is limited, the *FSHB* c.-211G>T SNP influences metaphase II oocytes, FOI, and OS duration. The most interesting findings are observed when multiple genotype analyses are performed, stressing the importance of multiple genetic analyses instead of focusing on a single SNP as performed in the previous papers (19, 21, 38). In detail, multivariate analysis revealed higher FOI and FORT together with specific LHCGR p.S312N and FSHR p.N680S haplotypes. Although this result is limited, it explains the variability of FOI and FORT in almost 10% of our cohort of patients.

Over the past years, our understanding of the role of SNPs in gonadotropins and their receptor genes, as well as their implications in the modulation of female reproduction, has significantly expanded. Nevertheless, the impact of SNPs on ART remains a subject of debate. Numerous studies involving women undergoing ART have attempted to assess whether the *FSHR* genotype could predict ovarian response and/or determine the starting FSH dose. This approach could lead to personalized OS, resulting in decreased incidence of hyper and poor response (4, 29), thereby conserving resources and preventing dropouts. The FSHR p.N680S SNP is one of the most investigated genetic variants in the IVF context (30). FSHR p.N680S S homozygosity has been correlated with reduced sensitivity to exogenous FSH (22), while the N allele with increased sensitivity to FSH (39). An *in vitro* study demonstrated that the pattern of FSH-dependent intracellular pathways are modulated according to the FSHR p.N680S genotype, and higher cAMP levels were achieved in homozygous asparagine than serine carriers (39, 40). From a clinical point of view, the reduced ovarian sensitivity is typically interpreted as the requirement of a higher total dose of gonadotropins used during OS (38, 41, 42), lower peak estradiol levels on the day of hCG administration (36, 43), a lower number of retrieved oocytes at pickup (16, 44), and higher basal FSH levels (16, 45). It was even suggested that the p.N680S N allele could be linked with higher risk of OHSS (39) and increased risk of progesterone rise (46) during OS, although these results should be confirmed by further studies. The evidence collected so far does not demonstrate any effect on pregnancy rate (16, 45). Our findings are consistent with the previous studies, confirming that the FSHR p.N680S N variant is linked to better duration of stimulation and ovarian sensitivity, rather than pregnancy and cumulative pregnancy rate. Notably, we detected that ovarian sensitivity, measured by the FOI index, is significantly high in women displaying specific combinations of FSHR p.N680S and LHCGR p.S312N SNPs. This observation supports that both LHCGR and FSHR play an important role during follicular recruitment and growth (47, 48). In contrast with previous studies, we observed for the first time that even serine carriers of FSHR p.N680S have better FOI than heterozygotes, only when they are also associated with serine in the LHCGR p.S312N. Instead, FORT was better in FSHR p.N680S N carriers. In other words, these data may suggest that the expression of the LHCGR p.S312N S variant could mitigate the reduced FOI but not the FORT index in the same way. This phenomenon might mean that LH actions might be more pronounced in oocyte output and maturation than follicular recruitment. The literature so far focuses on the role of these polymorphisms only in terms of the crude number of eggs retrieved that is not necessarily related to a good or a worse ovarian sensitivity (17). For instance, even a retrieval of a suboptimal number of eggs (3) (i.e., 4–9 eggs retrieved) could reflect a good ovarian sensitivity if the AFC is consistent (17). It is plausible that combined genetic variants could modulate the ovarian sensitivity rather than the number of eggs per se, stressing the importance of further and larger sample-sized study studies evaluating FOI and FORT as primary outcomes.

Previous studies demonstrated that the *FSHR* c.-29G>A SNP modulates the promoter transcriptional activity (36), and the G homozygosity was significantly associated with a higher number of collected oocytes (21, 24). More recently, it was observed that *FSHR* c.-29G>A homozygous carriers have reduced ovarian response (44), although other studies did not find any correlations (49, 50). In the present study, we did not detect any effect exerted by this polymorphism *per se* on OS. Rather, cumulative FSH dosage, FOI, FORT, and stimulation duration are modulated when the *FSHR* c.-29G>A SNP is combined with other genetic variants, suggesting that the evaluation of a single SNP could not have enough clinical relevance in predicting OS outcomes. Indeed, one of the main strengths of our investigation resides in the effort to analyze, prospectively, multiple genetic combinations associated with specific OS outcomes. Furthermore, for the first time we assessed parameters strictly related to ovarian sensitivity, such as FOI and FORT. Both could be effectively used to assess the efficiency of ovarian response in order to allow better tailoring of the treatment (51). The influence of genetic analysis on OS outcome could open a new scenario in personalization of protocols, avoiding unexpected reduced responses that affect a large number of women candidates for IVF (52). Of course, we recognized that such analysis required very large number of observations that certainly was not achieved by this multicentered study. In addition, with the purpose to limit biases, AFC evaluation was standardized among centers and a fixed FSH starting dose was maintained constant throughout the stimulation period. In this way, we overcome any possible limitation linked to adjustments in the FSH dose or prolonged treatment, which could mask the impact of genotypes on ovarian response (53, 54). Another important limitation is that the sample size was estimated only considering *FSHR* c.-29G>A, a genetic variant limiting the robustness of our findings regarding the other SNPs analyzed.

## Conclusion

In order to elucidate the role of gene SNPs in OS outcomes, we conducted a study in which the women’s setting was strictly established *a priori* to consider normo-responder women. In particular, the ovarian reserve has been considered among inclusion criteria, and the AFC was assessed using a reliable, standardized, automated method along with centralized AMH assessment. Furthermore, OS was administered with a fixed standard dose of 150 IU FSH daily.

Our study confirmed the presence of an association between specific genotype variants and OS outcomes. Indeed, separate analysis of gonadotropin and receptor SNPs revealed a mild association with OS outcomes. However, combined multivariate analysis of polymorphisms showed greater impact on the efficacy of OS outcomes, suggesting the potential role of these SNPs in the decision-making process of ART settings, regarding useful clinical quality indicators such as FOI (51). Obviously, multivariate analyses require a very large sample size to be considered as robust, and our findings should be corroborated by further studies. In the future, increasing use of real-world data and the support of artificial intelligence could offer the opportunity to dramatically increase the number of observations and thereby ameliorate the research in the field of pharmacogenomics, focusing more on the combination of genetic variants instead of single analysis.

Unfortunately, the clinical utility of a pharmacogenomic approach falls outside the scope of this prospective association analysis, despite a possible effect on ART outcome is being suggested. We hope that our findings could contribute in the future to the development of robust randomized controlled trials to demonstrate the clinical utility of a pharmacogenomic approach, focusing on FOI or FORT rather than endpoints such as pregnancy rate, which are affected by confounding factors, that is, the male factor.

## Data Availability

The raw data supporting the conclusions of this article will be made available by the authors, without undue reservation.
