# Supplementary material for: Impact of gonadotropin genetic profile and ovarian reserve on controlled ovarian stimulation: data from prospective cohort of the GENACOS trial
Source: Front Endocrinol (Lausanne). 2025 Aug 22;16:1601803. doi: 10.3389/fendo.2025.1601803 (PMC12411160; doi:10.3389/fendo.2025.1601803)
Supplement: Supplementary file 1 [file Table1.docx]

**Supplementary Table 1.** Genotypic association tests performed considering each SNP alone for the follicle output rate (FORT).

| **Model** | **Genotype** | **n** | **Response mean (SE)** | **Difference (95% CI)** | **P-value** |
| --- | --- | --- | --- | --- | --- |
| ***FSHR*** c.**-29G>A** | | | | | |
| **Codominant** | G/G | 53 | 1.07 (0.05) | 0.00 | 0.780 |
|  | G/A | 42 | 1.12 (0.07) | 0.05 (-0.11 – 0.21) |  |
|  | A/A | 10 | 1.15 (0.14) | 0.08 (-0.19 – 0.35) |  |
| **Dominant** | G/G | 53 | 1.07 (0.05) | 0.00 | 0.490 |
|  | G/A-A/A | 52 | 1.13 (0.06) | 0.05 (-0.10 – 0.21) |  |
| **Recessive** | G/G-G/A | 95 | 1.09 (0.04) | 0.00 | 0.670 |
|  | A/A | 10 | 1.15 (0.14) | 0.06 (-0.20 – 0.32) |  |
| **Overdominant** | G/G-A/A | 63 | 1.08 (0.05) | 0.00 | 0.660 |
|  | G/A | 42 | 1.12 (0.07) | 0.04 (-0.12 – 0.19) |  |
| **FSHR p.N680S** | | | | | |
| **Codominant** | N/N | 32 | 1.19 (0.09) | 0.00 | 0.270 |
|  | N/S | 51 | 1.05 (0.05) | -0.14 (-0.32 – 0.03) |  |
|  | S/S | 22 | 1.07 (0.07) | -0.12 (-0.33 – 0.10) |  |
| **Dominant** | N/N | 32 | 1.19 (0.09) | 0.00 | 0.110 |
|  | N/S-S/S | 73 | 1.06 (0.04) | -0.13 (-0.30 – 0.03) |  |
| **Recessive** | N/N-N/S | 83 | 1.11 (0.05) | 0.00 | 0.750 |
|  | S/S | 22 | 1.07 (0.07) | -0.03 (-0.22 – 0.16) |  |
| **Overdominant** | N/N-S/S | 54 | 1.14 (0.06) | 0.00 | 0.220 |
|  | N/S | 51 | 1.05 (0.05) | -0.09 (-0.25 – 0.06) |  |
| ***FSHB*** c.**-211G>T** | | | | | |
| **Codominant** | G/G | 76 | 1.13 (0.05) | 0.00 | 0.310 |
|  | G/T | 28 | 1.01 (0.07) | -0.12 (-0.29 – 0.05) |  |
|  | T/T | 1 | 1.38 (0.00) | 0.26 (-0.52 - -1.04) |  |
| **Dominant** | G/G | 76 | 1.13 (0.05) | 0.00 | 0.220 |
|  | G/T-T/T | 29 | 1.02 (0.07) | -0.11 (-0.28 – 0.06) |  |
| **Recessive** | G/G-G/T | 104 | 1.10 (0.04) | 0.00 | 0.470 |
|  | T/T | 1 | 1.38 (0.00) | 0.29 (-0.49 – 1.07) |  |
| **Overdominant** | G/G-T/T | 77 | 1.13 (0.05) | 0.00 | 0.160 |
|  | G/T | 28 | 1.01 (0.07) | -0.12 (-0.29 – 0.05) |  |
| **LHβ V-LH p.W8R** | | | | | |
| **Codominant** | W/W | 87 | 1.11 (0.04) | 0.00 | 0.630 |
|  | W/R | 18 | 1.06 (0.08) | -0.05 (-0.25 – 0.15) |  |
| **LHCGR p.S312N** | | | | | |
| **Codominant** | S/S | 45 | 1.09 (0.07) | 0.00 | 0.850 |
|  | N/S | 44 | 1.09 (0.05) | 0.00 (-0.16 – 0.17) |  |
|  | N/N | 16 | 1.15 (0.12) | 0.06 (-0.17 – 0.29) |  |
| **Dominant** | S/S | 45 | 1.09 (0.07) | 0.00 | 0.810 |
|  | N/S-N/N | 60 | 1.11 (0.05) | 0.02 (-0.15 – 0.17) |  |
| **Recessive** | S/S-N/S | 89 | 1.09 (0.04) | 0.00 | 0.570 |
|  | N/N | 16 | 1.15 (0.12) | 0.06 (-0.15 – 0.27) |  |
| **Overdominant** | S/S-N/N | 61 | 1.10 (0.06) | 0.00 | 0.870 |
|  | S/S | 45 | 1.09 (0.05) | -0.01 (-0.17 – 0.14) |  |
