# Supplementary material for: Impact of gonadotropin genetic profile and ovarian reserve on controlled ovarian stimulation: data from prospective cohort of the GENACOS trial
Source: Front Endocrinol (Lausanne). 2025 Aug 22;16:1601803. doi: 10.3389/fendo.2025.1601803 (PMC12411160; doi:10.3389/fendo.2025.1601803)
Supplement: Supplementary file 2 [file Table2.docx]

**Supplementary Table 2.** Genotypic association tests performed considering each SNP alone for the total number of embryos obtained.

| **Model** | **Genotype** | **n** | **Response mean (SE)** | **Difference (95% CI)** | **P-value** |
| --- | --- | --- | --- | --- | --- |
| ***FSHR*** c.**-29G>A** | | | | | |
| **Codominant** | G/G | 53 | 2.64 (0.23) | 0.00 | 0.190 |
|  | G/A | 43 | 2.74 (0.24) | 0.10 (-0.57 – 0.77) |  |
|  | A/A | 10 | 3.70 (0.63) | 1.06 (-0.07 – 2.19) |  |
| **Dominant** | G/G | 53 | 2.64 (0.23) | 0.00 | 0.390 |
|  | G/A-A/A | 53 | 2.92 (0.23) | 0.28 (-0.07 – 2.19) |  |
| **Recessive** | G/G-G/A | 96 | 2.69 (0.17) | 0.00 | 0.069 |
|  | A/A | 10 | 3.70 (0.63) | 0.28 (-0.36 – 0.92) |  |
| **Overdominant** | G/G-A/A | 63 | 2.81 (0.22) | 0.00 | 0.850 |
|  | G/A | 43 | 2.74 (0.24) | 1.01 (-0.07 – 2.09) |  |
| **FSHR p.N680S** | | | | | |
| **Codominant** | N/N | 32 | 2.78 (0.30) | **0.00** | 0.510 |
|  | N/S | 52 | 2.63 (0.22) | -0.15 (-0.89 – 0.59) |  |
|  | S/S | 22 | 3.14 (0.39) | 0.36 (-0.56 – 1.27) |  |
| **Dominant** | N/N | 32 | 2.78 (0.30) | 0.00 | 0.990 |
|  | N/S-S/S | 74 | 2.78 (0.20) | 0.00 (-0.70 – 0.70) |  |
| **Recessive** | N/N-N/S | 84 | 2.69 (0.18) | 0.00 | 0.270 |
|  | S/S | 22 | 3.14 (0.39) | 0.45 (-0.34 – 1.23) |  |
| **Overdominant** | N/N-S/S | 54 | 2.93 (0.24) | 0.00 | 0.370 |
|  | N/S | 52 | 2.63 (0.22) | -0.29 (-0.93 – 0.35) |  |
| ***FSHB*** c.**-211G>T** | | | | | |
| **Codominant** | G/G | 77 | 2.61 (0.17) | 0.00 | 0.200 |
|  | G/T | 28 | 3.21 (0.38) | 0.60 (-0.12 – 1.33) |  |
|  | T/T | 1 | 4.00 (0.00) | 1.39 (-1.90 – 4.68) |  |
| **Dominant** | G/G | 77 | 2.61 (0.17) | 0.00 | 0.085 |
|  | G/T-T/T | 29 | 3.24 (0.37) | 0.63 (-0.08 – 1.34) |  |
| **Recessive** | G/G-G/T | 105 | 2.77 (0.16) | 0.00 | 0.470 |
|  | T/T | 1 | 4.00 (0.00) | 1.23 (-2.09 – 4.54) |  |
| **Overdominant** | G/G-T/T | 78 | 2.63 (0.17) | 0.00 | 0.110 |
|  | G/T | 28 | 3.21 (0.38) | 0.59 (-0.13 – 1.31) |  |
| **LHβ V-LH p.W8R** | | | | | |
| **Codominant** | W/W | 88 | 2.76 (0.18) | 0.00 | 0.770 |
|  | W/R | 18 | 2.89 (0.40) | 0.13 (-0.73 – 0.98) |  |
| **LHCGR p.S312N** | | | | | |
| **Codominant** | S/S | 45 | 2.56 (0.26) | 0.00 | 0.330 |
|  | N/S | 45 | 3.07 (0.26) | 0.51 (-0.18 – 1.20) |  |
|  | N/N | 16 | 2.62 (0.31) | 0.07 (-0.89 – 1.03) |  |
| **Dominant** | S/S | 45 | 2.56 (0.26) | 0.00 | 0.230 |
|  | N/S-N/N | 61 | 2.95 (0.21) | 0.40 (-0.25 – 1.04) |  |
| **Recessive** | S/S-N/S | 90 | 2.81 (0.18) | 0.00 | 0.680 |
|  | N/N | 16 | 2.62 (0.31) | -0.19 (-1.08 – 0.71) |  |
| **Overdominant** | S/S-N/N | 61 | 2.57 (0.21) | 0.00 | 0.140 |
|  | S/S | 45 | 3.07 (0.26) | 0.49 (-0.15 – 1.14) |  |
